# Primary prostatic Burkitt’s lymphoma complicated with hemophagocytic lymphohistiocytosis: a case report and literature review

**DOI:** 10.3389/fonc.2025.1553415

**Published:** 2025-03-17

**Authors:** Xiong Guo, Wei Ai, Zhi Zhang, Zonglai Liu, Haibo Fu, Pan Gao, Fajun Liu

**Affiliations:** Department of Urology, The Second People’s Hospital of China Three Gorges University, The Second People’s Hospital of Yichang, Hubei, China

**Keywords:** prostatic cancer, Burkitt’s lymphoma, hemophagocytic, transurethral resection of the prostate, case report

## Abstract

This article reports a very rare case of primary prostatic Burkitt’s lymphoma. After transurethral resection of the prostate, the patient developed hemophagocytic lymphohistiocytosis, which rapidly progressed, leading to the patient’s death. Unfortunately, a definitive diagnosis was made only in the advanced stages of the disease, contributing to a delay in diagnosis and worsening of the patient’s condition. This report aims to improve the understanding of this disease and aid in its early recognition.

## Introduction

Burkitt’s lymphoma (BL) is an aggressive B-cell lymphoma originating from germinal center or post-germinal center B cells, strongly associated with c-Myc gene rearrangements and Epstein-Barr virus (EBV) infection. BL commonly presents as an extranodal malignancy or acute leukemia. According to the WHO classification, BL is categorized into three clinical subtypes: endemic, sporadic, and immunodeficiency-associated. Endemic BL primarily occurs in African children and is closely linked to EBV infection. Sporadic BL is found worldwide, with the central nervous system being the most frequently involved site. It can also affect the gastrointestinal tract, and bone marrow (1). Immunodeficiency-associated BL primarily occurs in individuals with HIV infection, as well as in recipients of allogeneic stem cell transplants or those with congenital immunodeficiency syndromes (2). Primary BL originating in the prostate is exceedingly rare. To date, only four cases of sporadic prostatic BL have been reported in the literature (3–6). Here, we report a case of primary prostatic BL in a patient admitted with urinary retention. After undergoing transurethral resection of the prostate (TURP), his condition rapidly deteriorated with hemophagocytic lymphohistiocytosis (HLH). Despite aggressive supportive care, the patient died three weeks after the operation.

## Case report

A 67-year-old male patient presented to our hospital with progressive dysuria for over four years, which had worsened in the past three hours. Four years ago, the patient developed dysuria, a weak urinary stream, frequency, urgency, and nocturia. He did not seek medical attention or undergo any kind of evaluation or treatment at that time. Two years ago, he experienced acute urinary retention and required urinary catheterization on two separate occasions. His symptoms improved significantly after conservative treatment with medication. Three hours prior to presentation, he experienced acute urinary retention again, accompanied by urine dribbling, without fever, night sweats, weight loss, or fatigue and presented to the hospital. The patient reported being in good health with no history of significant disease or surgery.

**Figure 1:**
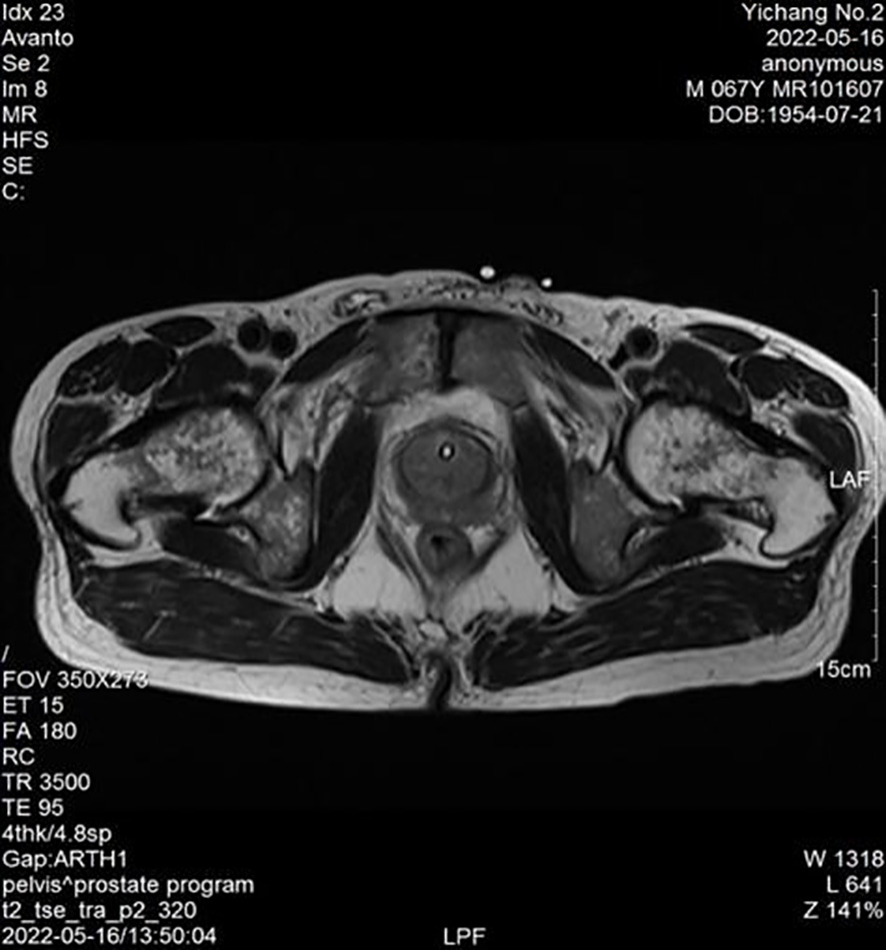
Prostate peripheral zone abnormal signals (MRI).

**Figure 2:**
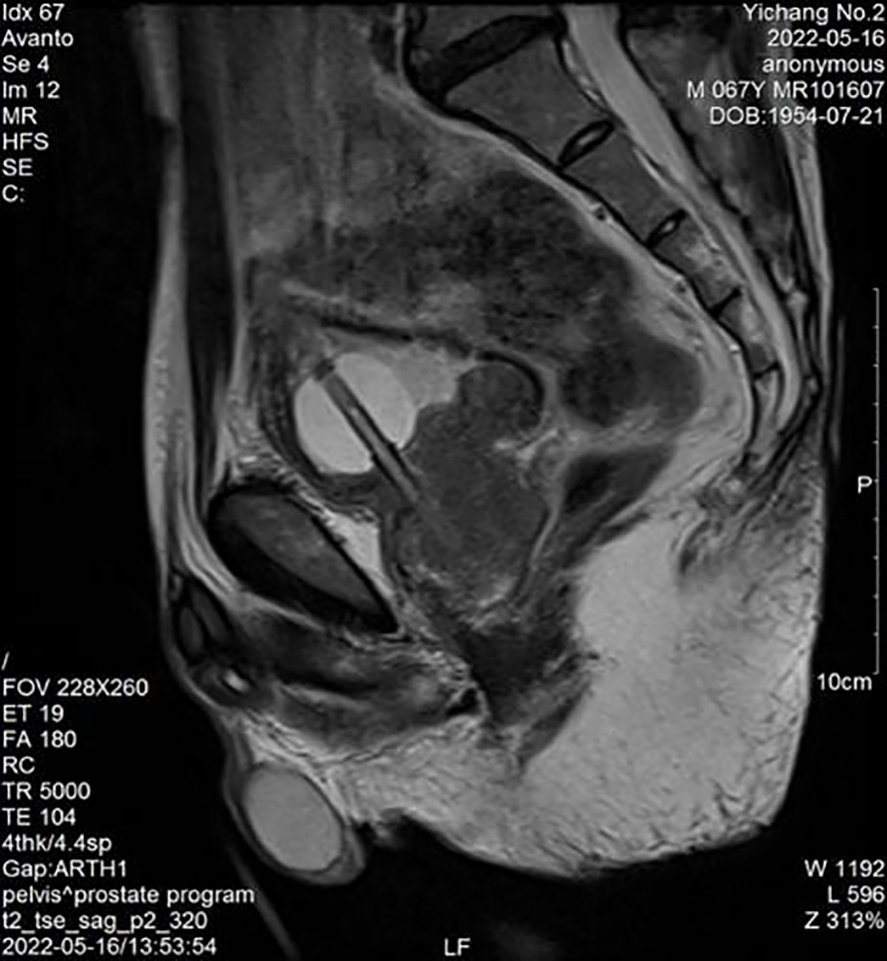
Prostate peripheral zone abnormal signals (MRI).

Physical examination: The lower abdomen showed distension and obvious tenderness. Digital rectal examination revealed a Grade 2 enlarged prostate with no nodules, normal texture, and a shallow central sulcus. Laboratory tests: Hemoglobin: 129 g/L; Platelet count: 78 × 10⁹/L; prostate-specific antigen (PSA): 0.93 ng/mL; free PSA (fPSA): 0.153 ng/mL; fPSA/PSA ratio: 0.16; prostatic acid phosphatase (PAP): 7.21 ng/mL, Triglyceride: 0.84 mmol/L and fibrinogen: 4.76 g/L. Liver and kidney function are normal. HIV antibody test is negative. Urine culture results were negative after three days. Prostate MRI revealed prostatic hyperplasia and abnormal signals in the peripheral zone, raising suspicion of prostate cancer with bladder invasion; diffusion-weighted imaging (DWI) was recommended (**Figures 1**, **2**). Bone ECT showed spotty foci of increased uptake in the bilateral pubis and ischium, suggesting active bone metabolism.

**Figure 3:**
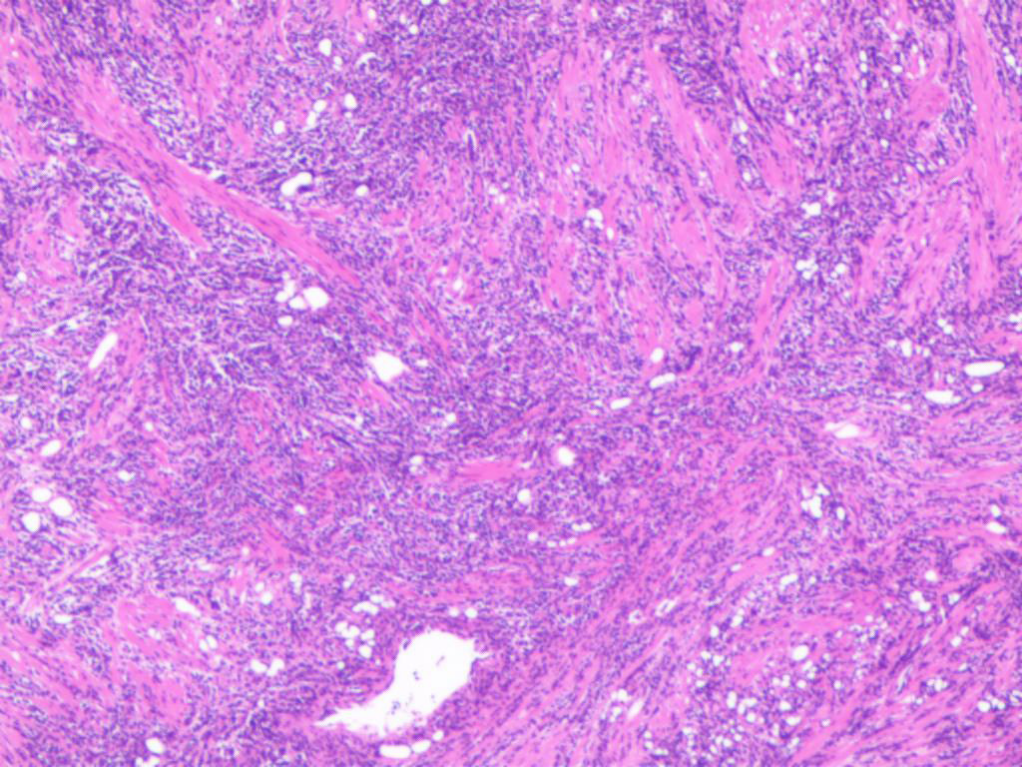
Diffuse distribution of tumor cells (HE*100).

**Figure 4:**
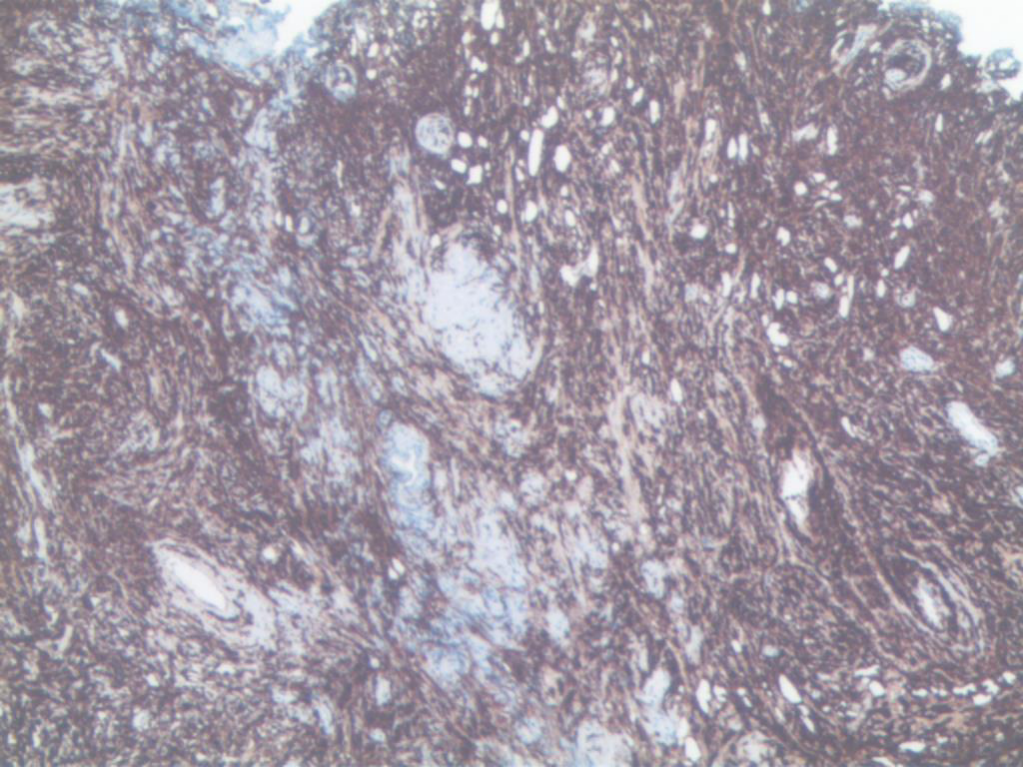
CD20 strongly positive of tumor cells (EnVision ×200).

The patient underwent TURP on May 21, 2022. Intraoperatively, the middle lobe of the prostate was notably hypertrophied, protruding into the bladder, with a friable texture and a tendency to bleed upon manipulation. Pathology revealed a non-Hodgkin B-cell lymphoma of the prostate. After excluding involvement of other sites, the diagnosis of primary prostatic lymphoma was established. Immunohistochemistry showed CD20(+), CD10(+), Bcl-6(+), P63(-), PSA(-), P504s(-), CK(H)(-), Ki67 (90%+), GATA-3(-), CD56(-), CgA(-), Syn(-), LCA(+), CK7(-), CK20(-), CD3(-), MUM1(-), CD21(-), Cyclin D1(-), and Bcl-2(-) (**Figures 3**, **4**). Further pathological review at a superior hospital confirmed the diagnosis of BL. Additional immunohistochemical findings included CD19(+), TCL1(+), LMO2(-), CD38(+), C-Myc (90%+), TdT(-), CD5(-), CD30(-), and P53 (weakly positive, 30%). *In situ* hybridization for EBV-encoded RNA (EBER) was negative. FISH analysis detected MYC gene rearrangement but no rearrangement of BCL2 or BCL6.

On May 29, the urinary catheter was removed, and the urine color was clear. A consultation by Department of Oncology recommended that further improvement of relevant examinations to determine the staging and systemic chemotherapy was advised. On June 1, the patient was transferred to the Department of Oncology and relevant examinations was carried out. Chest, abdomen, and pelvis contrast enhanced CT suggested prostatic hyperplasia with calcification, left kidney stone, left kidney cyst, right lung nodule (approximately 8mm) and left lung calcification. Brain, cervical, thoracic, lumbar MRI revealed bilateral lacunar cerebral infarctions in basal ganglia and cerebral white matter, cerebral atrophy, white matter degeneration, multiple abnormal signals in lumbar and sacrococcygeal vertebrae, bone CET examination was recommended. The ultrasonography of cervical, supraclavicular and axillary lymph nodes showed normal structural lymph nodes in the right axilla and bilateral neck, no lymph nodes detected in the bilateral supraclavicular region. A PET-CT scan was scheduled and further systemic chemotherapy was being prepared.

On June 2, the patient began to exhibit obvious gross hematuria and was immediately given continuous bladder irrigation. Routine blood tests were as follows: white blood cells (WBC) 13.3 × 10⁹/L, hemoglobin 94.8 g/L, platelets 15 × 10⁹/L, lactate dehydrogenase (LDH) 4232 U/L, and α-hydroxybutyrate 3560 U/L. Ultrasonography of the urinary tract showed a massive echogenic intravesical mass, suspected to be a large blood clot (approximately 800 mL in volume). Emergency transfusion of 1 unit of platelets, 2 units of red blood cells, and 800 mL of fresh frozen plasma and treatment with recombinant human thrombopoietin (rhTPO) and interleukin-11 were initiated. The patient was then transferred from the Department of Oncology to Department of Urology due to persistent prostatic bleeding. On June 4, the patient underwent open cystotomy and hematoma evacuation. A Hematology consultation was initiated, recommending platelet elevation, blood transfusion therapy, dynamic monitoring of blood routine and coagulation function.

On June 6, he developed a fever with a body temperature of 39.8°C. Routine blood tests revealed WBC 38.0 × 10⁹/L, hemoglobin 89 g/L, platelets 30 × 10⁹/L, interleukin-6 < 3 pg/mL, and procalcitonin 0.6 ng/mL. Considering the possibility of sepsis, broad-spectrum antibiotics were administered. On June 9, a multidisciplinary discussion, including hematology and oncology departments, was conducted for the patient. The discussion results concluded that bone marrow aspirate, trephine and peripheral blood cell morphology examination should be performed, followed by early chemotherapy after bleeding control. However, the patient refused further invasive diagnostics and opted for conservative treatment and the patient lost the chance of chemotherapy due to the persistent bleeding.

The patient continued to experience persistent hemorrhage of the prostate and uncontrolled fever, with hemoglobin and platelet levels continuing to decline. Repeated transfusions of 4 units of platelets, 15 units of red blood cells, and 800 mL of plasma were required. Broad-spectrum antibiotics, including cefoperazone-sulbactam, piperacillin-tazobactam, imipenem-cilastatin, and ornidazole, were repeatedly administered. However, prostatic hemorrhage and fever persisted without significant improvement. On June 15, he began to develop liver dysfunction, jaundice, and heart failure. Laboratory tests showed the following results: total bilirubin 107.9 µmol/L, direct bilirubin 77.3 µmol/L, indirect bilirubin 30.6 µmol/L, alanine aminotransferase (ALT) 137 U/L, aspartate aminotransferase (AST) 453 U/L, γ-glutamyl transferase (GGT) 897 U/L, total bile acids 74.7 µmol/L, fibrinogen 1.3 g/L, D-dimer 6530 ng/mL, creatinine 175 µmol/L, pro-BNP 6895 pg/mL, lactate dehydrogenase (LDH) 4232 U/L, and α-hydroxybutyrate 3560 U/L. Severe hyperlipidemia was also noted, with triglycerides at 6.56 mmol/L and serum ferritin at 1830 ng/mL. Blood culture results were negative after five days. A liver, bile, pancreas, and spleen color ultrasound revealed cholecystitis and splenomegaly. A hematology consultation raised suspicion of hemophagocytic lymphohistiocytosis (HLH), and a bone marrow biopsy was recommended again for further diagnosis. However, the patient once again refused further invasive diagnostics and opted for conservative treatment. On June 17, the patient began to develop agonal breathing, and electrocardiography indicated ventricular fibrillation. Cardiopulmonary resuscitation (CPR) was performed successfully. Further treatment was recommended, but the patient’s family refused, and he passed away after discharge (**Chart 1**).

## Discussion

Primary non-Hodgkin lymphoma of the prostate is an extremely rare entity in clinical practice, with most cases being secondary. Among primary non-Hodgkin B-cell lymphomas, diffuse large B-cell lymphoma (DLBCL) is the most common subtype, while Burkitt lymphoma (BL) is exceedingly uncommon. The currently accepted diagnostic criteria for primary prostatic lymphoma include the following: (1) The tumor is confined to the prostate and adjacent soft tissues; (2) There is no evidence of involvement of peripheral lymph node; (3)Systemic lymphoma is not identified within at least one month following the diagnosis of the primary tumor (7). The patient underwent a complete brain and spinal MRI, chest and abdominal contrast enhanced CT, superficial lymph node ultrasonography and bone ECT examination, but no evidence of tumor invasion was found, considering the primary nature of the disease. A PET-CT was scheduled, but the examination had to be abandoned due to the deterioration of the patient’s condition.

The primary clinical symptoms of prostatic lymphoma include lower urinary tract obstruction, pain, and hematuria, while PSA levels are usually normal or only mildly elevated. Consequently, many cases are preoperatively misdiagnosed as benign prostatic hyperplasia and are subsequently treated with TURP. Therefore, in younger male patients presenting with obstructive urinary symptoms, suspicious tumor signals on prostate MRI, and normal PSA levels, rare pathological types such as prostatic lymphoma should be considered. Another preoperative examination result showed low platelets (78× 10⁹/L), but we overlooked the importance of this clinical features and proceeded with TURP for the patient. However, the platelet count continued to decrease after surgery, and the postoperative prostatic bleeding was difficult to control. This increased the complexity of the patient’s treatment, and also this led to the delay of postoperative radiotherapy and chemotherapy for the tumor since we had to perform a series of interventions to control the bleeding.

BL cells express B-cell-associated antigens (CD19, CD20, CD22, CD79a) and germinal center markers (CD10 and Bcl-6). CD5, CD23, Bcl-2, and terminal deoxynucleotidyl transferase (TdT) are not expressed. The proliferation index, measured by Ki-67, can be close to 100%.Almost all BL cases have MYC gene translocations, most of which are t(8;14)(q24;q32), but no rearrangement of the BCL-2 or BCL-6 genes (8).

Hemophagocytic lymphohistiocytosis (HLH) is a hyperinflammatory syndrome caused by genetic or acquired immune dysregulation, leading to a uniform clinical presentation. The excessive proliferation and activation of CD8 T lymphocytes and macrophages, as well as the development of a cytokine storm, are recognized as central pathophysiological mechanisms (9). HLH is classified into two types based on etiology: primary (genetic) and secondary (acquired). Primary HLH mainly occurs in children, with over 90% of cases presenting before the age of 2, often accompanied by a positive family history. It is associated with mutations in genes such as PRF1, UNC13D, STX11, STXBP2, RAB27A, CHS1/LYST, AP3B1, SH2D1A, and BIRC4 (10). In contrast, secondary HLH is more common in adults and is triggered by factors such as infections, malignancies, autoimmune diseases, acquired immunodeficiencies, drugs, and transplantation (9, 11).Among secondary causes, infections and malignancies are the most common. In the malignancy-associated HLH(M-HLH) group, lymphoma represent the most common trigger with peripheral T-cell lymphoma and NK/T-cell lymphoma being the primary contributors (11–13) B-cell lymphoma-associated HLH is less frequent, with diffuse large B-cell lymphoma being the most common subtype (7). The mechanisms by which extranodal lymphomas predispose individuals to HLH remain unclear. Several plausible mechanisms have been suggested. First, malignant cells can cause persistent antigenic stimulation and hypersecretion of pro-inflammatory cytokines. Second, genetic immune deficiencies, such as HAVCR2 mutations and X-linked lymphoproliferative disease, may predispose individuals to both cancer and HLH. Third, loss of immune homeostasis due to factors like chemotherapy, immune-activating therapies, hematopoietic stem cell transplantation, or infection drives this iatrogenic form of M-HLH (9). In this case, the patient experienced progressive platelet decline and uncontrollable fever, which was initially misdiagnosed as sepsis leading to concerns about antibiotic use. The rapid clinical deterioration after TURP, followed by subsequent laboratory tests confirming HLH activation. The pathological mechanism of HLH activation after surgery remains worth exploring. However, it is considered that TURP for prostate lymphoma is inappropriate; a prostate biopsy would be more suitable for diagnosing this disease.

The most common clinical features of HLH include fever, hepatosplenomegaly, progressive pancytopenia, hyperferritinemia, hypertriglyceridemia, hypofibrinogenemia, and multiorgan dysfunction. Hepatic dysfunction, coagulopathy, and various neurological symptoms are also frequently observed (14). Since HLH represents a wide spectrum of hyperinflammatory disorders with heterogeneous inciting conditions, and the diagnosis can be challenging because malignancies can also lead to elevated associated parameters. The HLH-2004 (15) diagnostic criteria require one of the following two conditions for diagnosis:①Molecular evidence supporting HLH. ②Fulfillment of five out of eight clinical and laboratory criteria: fever lasting more than one week with peaks >38.5°C; splenomegaly; cytopenias involving at least two cell lines (hemoglobin <90 g/L, platelets <100 × 10⁹/L, and absolute neutrophil count <1.0 × 10⁹/L); hypertriglyceridemia (>3 mmol/L) or hypofibrinogenemia (<1.5 g/L); hemophagocytosis observed in bone marrow or lymph nodes; decreased or absent NK cell activity; elevated ferritin levels (>500 ng/mL); and elevated soluble CD25 (sIL-2 receptor >2400 U/mL). Our patient fulfilled five criteria. NK cell activity and soluble CD25 were not performed due to technical limitations. Furthermore, hemophagocytosis was not detected because patients refused bone marrow aspirate and trephine. According to the HLH-2004 criteria, evidence of hemophagocytosis is not necessary for the diagnosis of HLH, and it should never be diagnosed or excluded solely based on the presence or absence of hemophagocytosis (15, 16). Several studies have shown that tissue hemophagocytosis is frequently observed in the absence of HLH; with infections, blood transfusions, autoimmune disease, and bone marrow failure (17). However, it has been argued that the HLH-2004 criteria are better suited for primary HLH and may not apply effectively to secondary HLH. In secondary HLH, patients often fail to meet five or more criteria during the early disease stages (e.g., hemophagocytosis). In particular, soluble IL-2 receptor and NK-cell activity are difficult to assess in most institutions. These diagnostic limitations can delay recognition and treatment until the disease has advanced. In a retrospective analysis using the MD Anderson Cancer Center database, only 21% of patients with suspected HLH met HLH-2004 diagnostic criteria (18). In 2009, Alexandra H. Filipovich (19) proposed a modified diagnostic approach, which considered three of the four criteria—fever, splenomegaly, cytopenias involving at least two cell lines, and hepatitis—combined with at least one of the following laboratory findings: hemophagocytosis, elevated ferritin, increased sCD25, or reduced NK cell activity. Fardet L et al. (20) developed a diagnostic scoring system, HScore, by which the likelihood of HLH is calculated based on ten clinical and laboratory parameters. The optimal cutoff value for HScore was 169, corresponding to a sensitivity of 93% and a specificity of 86%. Based on the H-score, our patient scored 209 with a >88% probability of HLH. Zoref-Lorenz A et al. (21) introduced a new tool, the optimized HLH inflammatory (OHI) index, which combines sCD25 >3900 U/mL and ferritin >1000 ng/mL to provide a simplified and accurate diagnostic and prognostic tool. Their study identified a large group of patients with high mortality risk who were not classified as HLH based on HLH-2004/HScore but were identified based using the OHI index. Gevorg N. Tamamyan et al. (18) collected data on 18 variables to aid in the diagnosis of HLH. Patients who met 5 of the 18 above-mentioned criteria would be considered to have a high probability of secondary HLH, improving the early diagnosis of HLH. According to these diagnostic criteria, Our patient fulfilled 12 criteria, making highly likely of HLH. The HLH-2004 diagnostic criteria were developed for children with familial HLH. Meanwhile, the HScore has limitation because sCD25 results were not included in the score and people with autoimmune/autoinflammatory-triggered HLH were not involved in the study. A multicenter study designed to optimize and validate diagnostic criteria for a heterogeneous cohort of secondary HLH patients found that the original HLH-2004 criteria, with a lowered cut-off of four fulfilled criteria was the best performing HLH diagnostic criteria set over all validation cohorts, followed by revHLH-2004(NK cell activity removed) and an HScore of 169 as a cut-off. However, the sIL-2R and OHI index performed inferior over all validation cohorts (22). A single-center, prospective cohort study of adults with secondary HLH suggested that the diagnostic performance of HLH-2004 criteria in adults is comparable to that in pediatric patients. However, the diagnostic performance of the H-score was lower in patients with malignancy-associated HLH. In their study, the performance of soluble CD25 may have been overestimated, as more than half of the patients had lymphoid malignancies, and where soluble CD25 is often elevated in lymphomas (23). In this case, the patient developed persistent high-grade fever postoperatively, with normal levels of IL-6 and procalcitonin, unresponsive to multiple adjustments in antibiotic regimens. Concurrently, unexplained progressive platelet decline and persistent bladder bleeding emerged, necessitating repeated blood transfusions and erythropoietin (EPO) therapy. Only after the patient presented with jaundice, hepatic failure, heart failure, and disseminated intravascular coagulation (DIC) was the diagnosis of HLH confirmed. Unfortunately, this delay in diagnosis significantly impacted the patient’s prognosis.

Malignancy-associated HLH progression is rapid, and has a high fatality rate. The median survival was only 1.5 months (18). Patients typically succumb to DIC, hemorrhage, infection, or multi-organ failure. The treatment of HLH is mainly divided into two phases: controlling excessive inflammation (remission induction) and replacing the defective immune system (allogeneic hematopoietic stem cell transplantation [allo-HSCT]). The Histiocyte Society introduced the first international treatment standard for first phases for HLH in 1994 (24), reporting a survival rate of 55% with a median follow-up of 3.1 years. It remains the first-line treatment for controlling acute inflammation in HLH. The HLH-94 treatment standard includes combination therapy with dexamethasone, cyclosporin A, and etoposide. Subsequently, the HLH-2004 trial suggested that adding cyclosporine to the HLH-94 regimen did not help control acute immune activation (25). In 2008, Shin et al. (26) directly applied the classical protocol for treating lymphoma (the CHOP protocol) to treat adult HLH, demonstrating its efficacy in alleviating lymphoma-associated HLH. Allo-HSCT remains to be the only potentially curative treatment for HLH after achieving complete remission (CR) or partial remission (PR) (14). In 20-30% of adult cases, HLH is refractory to first-line treatment or relapses after initial remission (27). The pathogenesis and development of HLH involve a range of pro-inflammatory cytokines, such as IFN-γ, IL-1β, IL-6, IL-18, and TNF-α. Several targets have been proposed in recent years, including IFN-γ, IL-6, TNF-α, IL-1, IL-18, CD52, CD20 and programmed cell death protein 1 (PD-1). In 2018, emapalumab, the monoclonal antibody targeting IFN-γ, gained global approval for the treatment of HLH, which mark the beginning of targeted therapies for HLH (28). Other agents such as the IL-1 receptor antagonist anakinra, the IL-6 inhibitor tocilizumab, and the IL-18 inhibitor recombinant human IL-18BP, have shown promising clinical results (29–31). The Janus kinase (JAK) inhibitor ruxolitinib has also shown promising therapeutic outcomes in recent studies, which suggest that JAK-STAT inhibition can reduce immune hyperactivation and improve patient outcomes (32). Additionally, emerging evidence suggests that PD-1 inhibitors hold potential for treating HLH, particularly in cases secondary to lymphoma (33). However, further clinical trials are needed to validate the efficacy and safety of these agents.

## Conclusion

Primary prostate lymphoma is extremely rare in clinical practice and difficult to diagnose, typically being identified only after postoperative pathological examination. When combined with hemophagocytic lymphohistiocytosis (HLH), the disease progresses rapidly, and the mortality rate is extremely high. Unexplained postoperative fever and progressive pancytopenia should prompt early screening for HLH-related markers, allowing for timely diagnosis and intervention.

## Data Availability

The original contributions presented in the study are included in the article/**Supplementary Material**, further inquiries can be directed to the corresponding author/s.

## Supplementary material

Chart 1 Diagnosis and treatment flow chart.
