# Supplementary figures and images for: Primary prostatic Burkitt’s lymphoma complicated with hemophagocytic lymphohistiocytosis: a case report and literature review
Source: Front Oncol. 2025 Mar 17;15:1553415. doi: 10.3389/fonc.2025.1553415 (PMC11955490; doi:10.3389/fonc.2025.1553415)

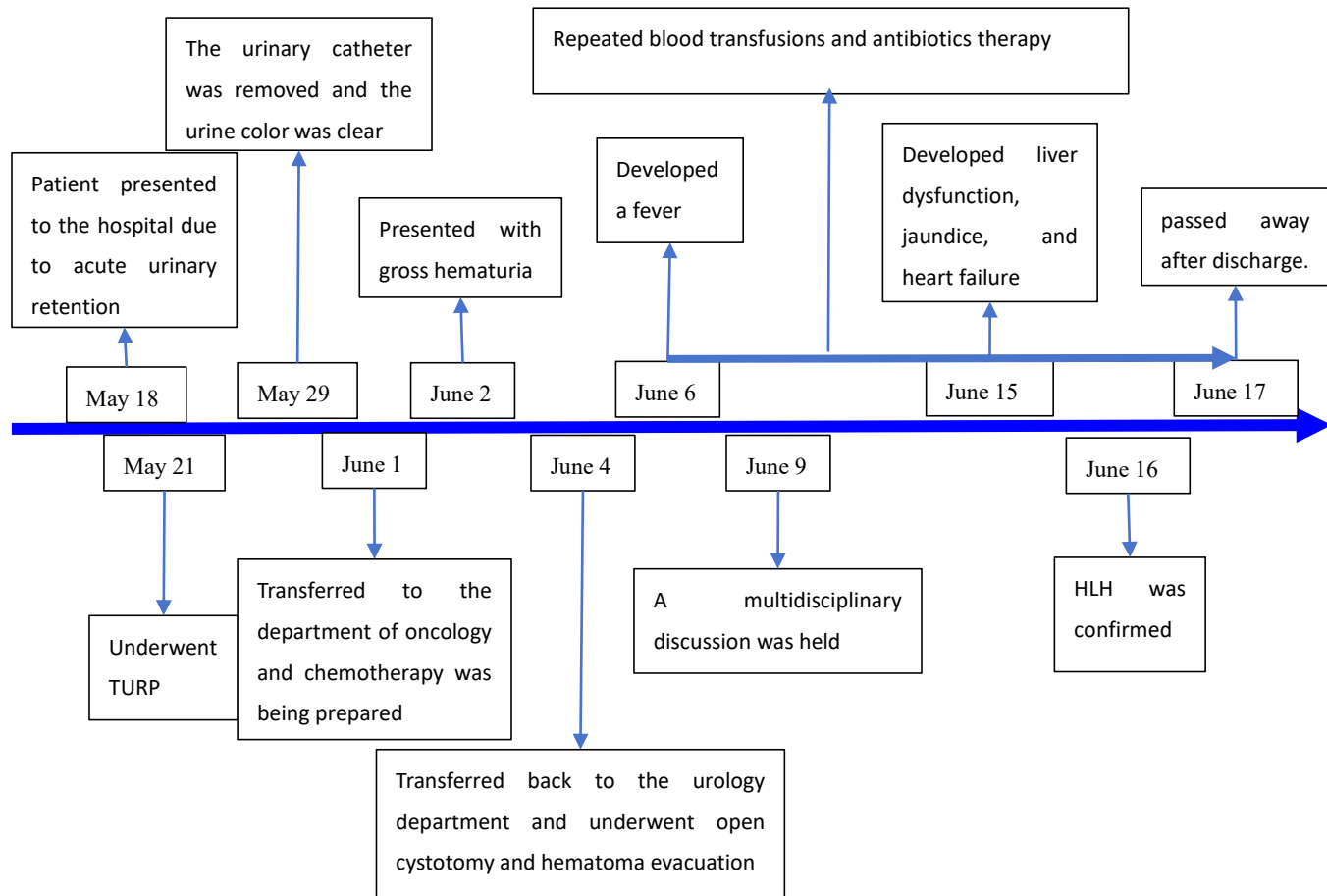

Supplement: Chart 1 — Diagnosis and treatment flow chart. [file DataSheet1.pdf]
